# Genetic profiling of putative breast cancer stem cells from malignant pleural effusions

**DOI:** 10.1371/journal.pone.0175223

**Published:** 2017-04-19

**Authors:** Verena Tiran, Stefanie Stanzer, Ellen Heitzer, Michael Meilinger, Christopher Rossmann, Sigurd Lax, Oleksiy Tsybrovskyy, Nadia Dandachi, Marija Balic

**Affiliations:** 1 Department of Internal Medicine, Division of Oncology, Medical University of Graz, Graz, Austria; 2 Institute of Human Genetics, Medical University of Graz, Graz, Austria; 3 Department of Internal Medicine, Division of Pulmonology, Medical University of Graz, Graz, Austria; 4 Second Internal Division of Pulmonology, Otto Wagner Spital, Vienna, Austria; 5 Institute of Pathology, LKH Graz West, Graz, Austria; 6 Institute of Pathology, Medical University of Graz, Graz, Austria; 7 Research Unit Epigenetic and Genetic Cancer Biomarkers, Medical University of Graz, Graz, Austria; 8 Research Unit Circulating Tumor Cells and Cancer Stem Cells, Medical University of Graz, Graz, Austria; Università degli Studi della Campania "Luigi Vanvitelli", ITALY

## Abstract

A common symptom during late stage breast cancer disease is pleural effusion, which is related to poor prognosis. Malignant cells can be detected in pleural effusions indicating metastatic spread from the primary tumor site. Pleural effusions have been shown to be a useful source for studying metastasis and for isolating cells with putative cancer stem cell (CSC) properties. For the present study, pleural effusion aspirates from 17 metastatic breast cancer patients were processed to propagate CSCs *in vitro*. Patient-derived aspirates were cultured under sphere forming conditions and isolated primary cultures were further sorted for cancer stem cell subpopulations ALDH1^+^ and CD44^+^CD24^-/low^. Additionally, sphere forming efficiency of CSC and non-CSC subpopulations was determined. In order to genetically characterize the different tumor subpopulations, DNA was isolated from pleural effusions before and after cell sorting, and compared with corresponding DNA copy number profiles from primary tumors or bone metastasis using low-coverage whole genome sequencing (SCNA-seq). In general, unsorted cells had a higher potential to form spheres when compared to CSC subpopulations. In most cases, cell sorting did not yield sufficient cells for copy number analysis. A total of five from nine analyzed unsorted pleura samples (55%) showed aberrant copy number profiles similar to the respective primary tumor. However, most sorted subpopulations showed a balanced profile indicating an insufficient amount of tumor cells and low sensitivity of the sequencing method. Finally, we were able to establish a long term cell culture from one pleural effusion sample, which was characterized in detail. In conclusion, we confirm that pleural effusions are a suitable source for enrichment of putative CSC. However, sequencing based molecular characterization is impeded due to insufficient sensitivity along with a high number of normal contaminating cells, which are masking genetic alterations of rare cancer (stem) cells.

## Introduction

Breast cancer is the most frequently diagnosed malignancy in females and the second leading cause of cancer-related deaths [1]. Despite the recent progress in early detection and treatment, breast cancer mortality remains high mainly due to cancer metastases. Thus, there is an urgent need to establish novel and even more effective treatment strategies.

We and others have demonstrated that cancer cells spread early during the course of disease and this micrometastatic spread is regarded prerequisite to overt metastasis and it is the rationale behind the use of adjuvant systemic treatment in patients when the primary tumor is removed and no metastatic spread can be detected at routine staging [2, 3]. In order to establish more effective treatment strategies, it is imperative to better understand the biology of metastasis and metastatic cells.

There is a large body of evidence describing stem cells in normal tissues [4, 5]. These cells are defined by their trait of self-renewal through asymmetrical cell division, generating both stem cells and committed progenitor cells [6]. This cellular and functional heterogeneity and hierarchical organization has not only been shown for normal tissues, but has also been demonstrated throughout a variety of cancer studies [7, 8]. At least to a certain extent, this observation has been associated with the presence of cellular hierarchy within tumors assembling the one in tissues. In the recent decade there is also increasing evidence indicating the presence of a subpopulation of tumor cells associated with stem cell properties, self-renewal, generation and maintenance of tumors [9]. These cells are supposed to be derived either from stem cells of normal tissues or from cells further downstream in development, which regained the properties of stem cells, like self-renewal and differentiation potential. The cancer stem cell (CSC) model has been applied to both hematologic malignancies [10] and solid tumors and it has been made at least partly responsible for tumor resistance and treatment failure [11]. There is solid evidence demonstrating the presence of breast cancer stem cells [4] [12].

Early studies demonstrating the presence of breast cancer stem cells mostly used malignant pleural effusions as a metastatic source to isolate and enrich for breast cancer stem cells. For example, Al-Hajj et al used pleural effusions to identify CSCs with the CD44^+^CD24^-/low^ phenotype with increased tumorigenicity and self-renewal property [13]. They showed that only 100 cells with the according phenotype generated tumors in mice, whereas thousands of cells with other phenotypes failed to do so. Consequently, the CD44^+^/CD24^low/-^ subpopulation has been characterized as highly tumorigenic and capable of self-renewal and differentiation [14]. The CD44 cell surface marker plays an important role in tumor cell proliferation and takes a key part in metastatic processes such as motility, migration and invasion [15–17]. In contrast, CD24 expression leads to an inhibition of cell migration via the SDF-1 cascade [18].

Another marker has also emerged as a putative stem cell marker for normal tissues and cancer, particularly breast cancer, namely aldehyde dehydrogenase 1 (ALDH1) [19]. ALDH1 is a detoxifying enzyme responsible for the oxidation of intracellular aldehydes and seems to have a role in early differentiation of stem cells through its role in oxidizing retinol to retinoic acid [20]. The expression of ALDH1 has been used in flow cytometry sorting and cells with its expression have been highly enriched for putative breast cancer stem cells. Further, its expression in breast cancer tissues has been correlated with worse prognosis [21, 22]. In later studies, ALDH1 was also identified as an enzyme active in breast cancer cells associated with stem cell properties and isolated from malignant pleural effusions [23].

The advantage of using pleural effusions is that patients have to undergo the thoracentesis for therapeutic purposes in order to achieve relief of symptomatic dyspnea. Thus, this invasive procedure is not being performed for the purposes of the present study. This fact along with the evidence of a significant percentage of breast cancer stem cells in pleural effusions lead to the present study with the focus on evaluating pleural effusions as a source for enrichment of described putative breast cancer stem cell phenotypes and the subsequent molecular characterization [24, 25]. We generated patient-derived cell cultures and investigated molecular differences of CSC associated subpopulations as a potential for establishing biomarkers associated with breast cancer stem cell phenotypes.

## Materials and methods

### Patient samples

Malignant pleural effusions were collected from breast cancer patients undergoing thoracentesis for treatment of symptomatic dyspnea at the Division of Pulmonology, Department of Internal Medicine, Medical University of Graz, Austria between September 2011 and April 2013. Patients with histologically verified malignant breast cancer and age between 18 and 90 years were eligible for the study. The study was approved by the local ethics committee of the Medical University of Graz (EK Nr. 20–309 ex 08/09) and a signed written informed consent was obtained from all patients. All patient information was anonymized and identifiers were removed prior to analysis. This study was of explorative design and thus no statistical power analysis was performed. Altogether, 20 patients were included in this study, from which 17 had the confirmed breast cancer, and 3 were diagnosed with concomitant second metastatic cancer. Where available, samples of corresponding primary tumors and/ or metastasis were matched and analyzed, as described below.

### Cell isolation from pleural effusions aspirates

The first 8 pleural effusion samples were diluted 1:1 with Dulbecco′s modified eagle media (DMEM) low glucose (PAA Laboratories GmbH, Pasching, Austria), 10% fetal bovine serum (FBS gold PAA Laboratories GmbH, Pasching, Austria) and 2% antibiotic-antimycotic (ABAM Gibco, Thermo Fischer Scientific, MA, USA). The isolation procedure for samples PL13-25 was optimized, whereby the pleural effusion aspirates were filtered through a wide-meshed strainer at the beginning. The volume processed varied between 10 mL and 1500 mL, depending on the amount of the obtained material. After a centrifugation step at 400xg for 15 min, the cells were washed with phosphate buffer saline (PBS) and centrifuged at 800xg for 15 min. The cell suspension was treated with an ammonia lysis buffer for 5 minutes. After the lysis step, the cell suspension was filtrated through a 70 μm filter. Cells were counted, and a median number of 40.3*10^7^ cells were transferred to culture (range: 4.3*10^6^−3*10^9^). Cells were seeded in ultra-low attachment flasks (Corning, New York, USA) and sphere formation assay was initiated. Alternatively, cells were seeded in adherent cell culture with DMEM low glucose, 10% FBS and 2% ABAM. The cells were grown at 37°C and 5% CO_2_ conditions.

### Sphere formation assay

The sphere formation assay was performed according to a previous publication [26]. Briefly, cells were seeded and grown in serum-free Mammary Epithelial Basal medium (MEBM, Lonza, Basel, Switzerland), supplemented with 10 ng/mL basic fibroblast growth factor (bFGF), 20 ng/mL epidermal growth factor (EGF), 5 μg/mL insulin (both from Peprotech, New York, USA), and 20 μL/mL B27 supplement (Invitrogen, Leek, Netherlands). After the first passage, mammospheres were filtered through a 40 μm Nylon Cell Strainer (BD, Falcon) to obtain purer spheres for further culture. Thereafter, cells were dissociated with Accutase (PAA Laboratories), following incubation at 37°C for 4 min. Cells were washed with two volumes of PBS to inactivate the enzyme, resuspended in MEBM containing supplements and seeded for generation of secondary spheres.

### Flow cytometry and cell sorting

For flow cytometry analysis cells were isolated and dissociated with Accutase (PAA Laboratories) were then blocked with Blocking Buffer, consisting of horse serum 1:20 in 6% bovine serum albumin/PBS, for 20 min at 4°C. The staining of the cells for further analysis was performed according to two different protocols, as adapted from our previous publication [3]. Briefly, for the first staining 1*10^6^ cells were taken and incubated with aliquots of antibodies (2.5 μL anti-CD44 Allophycocyanin and 5 μL anti-CD24 Fluorescein isothiocyanate, BD Bioscience, Schwechat, Austria) for 30 minutes at 4°C. For the depletion of hematopoietic progenitor cells, staining with Phycoerythrin labeled lineage marker cocktail containing anti-CD2, -CD3, -CD10, -CD16, -CD18, -CD31, -CD64, and -CD140b (all BD Biosciences) [13] was used. Another approach was to measure the ALDH1 activity with Aldefluor assay kit (Stem Cell Technologies, Grenoble, France), performed according to the manufacturer instructions. The monoclonal antibodies were all pretitered to determine their optimal dilutions before use. Flow cytometry sorting was performed on the fluorescence activating cell sorter (FACS) Aria (BD Bioscience). Cells without staining, single stained cells and isotype controls (BD Bioscience) were used as controls in the experiments. Side scatter and forward scatter profiles were used to eliminate cell doublets and apoptotic cells were excluded by using 7-aminoactinomycin D (BD Bioscience). Data analysis was performed using the Diva software 7.0 (BD Bioscience). Sorted cells were seeded into ultra-low attachment flasks for evaluation of sphere formation capacity and DNA/RNA was extracted for sequencing purposes.

### DNA extraction

Genomic DNA from sorted cells was extracted with the QIAamp DNA Micro Kit (Qiagen, Hilden, Germany) according to the manufacturer’s protocol with the addition of 5 ng/μL carrier RNA per sample. DNA was dissolved in a final volume of 30 μL deionized water and the concentration was measured spectrophotometrically with Nanodrop 2000 (Thermo Scientific; MA USA).

### Short Tandem Repeat (STR) analysis

For STR analysis 0.7 ng of extracted DNA were amplified with the PowerPlex 16HS System (Promega, Mannheim, Germany) according to manufacturer´s instruction on a thermocycler MyCylcer (Biorad, Vienna, Austria). In this analysis 16 STR loci can be evaluated such as Penta E, D18S51, D21S11, TH01, D3S1358, FGA, TPOX, D8S1179, vWA, Amelogenin, Penta D, CSF1PO, D16S539, D7S820, D13S317 and D5S818. The amplified fragments were detected with a capillary electrophoresis on the 3730 Genetic Analyzer (Applied Biosystem, Vienna, Austria).

### Copy number profiling

Genome wide copy number aberrations (CNA) were established using low-coverage whole genome sequencing. Shotgun libraries were prepared using the TruSeq DNA Nano LT Sample Preparation Kit (Illumina, San Diego, CA, USA) with slight modifications to the manufacturer’s protocol. Depending on the DNA concentrations, 50–100 ng of DNA from sorted cell fractions and 1–2 μg of DNA from tumor samples were fragmented in 130 μL using the Covaris System (Covaris, Woburn, MA, USA). After concentrating the volume to 50 μL end repair, A-tailing and adapter ligation were performed following the manufacturer’s instructions. For selective amplification of the library fragments that have adapter molecules on both ends we used 8–15 PCR cycles. Libraries were quality checked on an Agilent Bioanalzyer using a DNA 7500 Chip (Agilent Technologies, Santa Clara, CA, USA) and quantified using qPCR with a commercially available PhiX library (Illumina) as a standard. Six libraries were pooled equimolarily and sequenced on an Illumina MiSeq in a 150 bp single read run. On the completion of the run data were base called, demultiplexed on the instrument (provided as Illumina FASTQ 1.8 files, Phred+33 encoding), and FASTQ format files in Illumina 1.8 format were used for downstream analysis. Copy number analysis was performed as previously described [27]. Briefly, low-coverage whole-genome sequencing reads were mapped to the pseudo-autosomal-region (PAR)-masked genome and reads in different windows were counted and normalized by the total number of reads. We further normalized read counts according to the GC-content using LOWESS-statistics. In order to avoid position effects we normalized the sequencing data with GC-normalized read counts of a set of 30 non-malignant control samples [27]. Subsequently we generated segments of similar copy-number values by applying circular binary segmentation (CBS) and Gain and Loss Analysis of DNA (GLAD). All sequencing raw data were deposited at the European Genome-phenome Archive (EGA, http://www.ebi.ac.uk/ega/), which is hosted by the EBI, under the accession number EGAS00001002343.

### Multicolor Immunofluorescence

The expression of selected breast cancer related proteins was analyzed with multicolor immunofluorescence staining. Therefore, 4 μm thick sections from compact spheres that were priorly formalin-fixed and paraffin-embedded were used. After retrieval with high pH solution (Dako, Glostrup, Denmark) in the microwave at 360 W for 10 min and blocking with normal goat serum for 30 min, samples were incubated with primary monoclonal antibodies such as rabbit anti-pan-cytokeratin, mouse anti-Ki67, mouse anti-Vimentin, mouse-anti human epithelial antigen (HEA), rabbit anti-Her2neu (all Dako) and mouse anti-ALDH1 (BD Bioscience) and mouse anti-CD44 (Thermo Scientific) for 1 hour at room temperature. After washing with PBS, slides were incubated for 1 hour with secondary fluorescent labeled antibody cocktail consisting of Alexa 488 goat anti-rabbit IgG and Alexa 594 goat anti-mouse IgG (life technologies, Carlsberg, CA, USA). Cells were washed again and slides were coverslipped with the SlowFade^®^ gold antifade mounting media with 4′,6-Diamidin-2-phenylindol (DAPI) (life technologies). Image analysis was performed with a fluorescent microscope Olympus Basic BX51 (Vienna, Austria).

## Results

### Characteristics of study participants

In total, 20 patients were included in this study. After careful revision of medical and pathological records, three patients were excluded from the study due to the origin of the pleural effusion other than breast cancer. The first four samples (PL1 to PL4) were used to establish and optimize the protocol for cell culture, but results were not included into this study. From one patient two pleural samples were available (sample no PL16 and PL22). Altogether, 18 pleural samples from 17 patients (85%) were completely analyzed.

Table 1 summarizes clinical and pathological data of all 17 patients analyzed. All patients included had metastatic breast cancer, with a median age of 71 (range 49–91). 15 out of 17 patients (83%) were diagnosed with an invasive ductal carcinoma of the breast (according to the new classification NST). A positive ER status was found in 11/17 samples (64%) and a positive PR status in 9/17 samples (53%). 8/17 patients (47%) had Her2neu positive breast cancer. In 8 cases, additional tumor material was available (primary tumor, one bone metastasis, one pleural effusion cyto block).

**Table 1:** Summary of clinical and pathological data from 17 patients.

Sample no.	Age	ER/PR	Her2	Ki67	Metastasis	Diagnosis
PL5	70	n.i.	n.i.	n.i.	n.i.	invasive ductal
PL6	87	pos/neg	3+	16%	bone.	invasive ductal
PL7	91	pos/pos	neg	n.i.	none	invasive ductal
PL8	69	pos/pos	neg	n.i.	lymph node and bone	invasive ductal
PL9	85	pos/pos	2+	n.i.	lymph node and bone	Invasive ductal
PL10	84	neg/neg	neg	70%	lymph node	invasive ductal
PL11	48	neg/neg	pos	n.i.	liver, bone, brain and lymph node	invasive ductal
PL12	56	neg/neg	neg	70%	lung and brain	invasive ductal
PL13	76	moderately pos/ moderately pos	neg	25%	lung	invasive ductal
PL15	49	neg/neg	2+	90%	lymph node	invasive lobular
PL16/PL22	56	pos/pos	neg	n.i.	lymph node	invasive ductal
PL17	77	highly pos/ moderately pos	neg	n.i.	lung	invasive ductal
PL19	77	pos/pos	neg	n.i.	n.i.	invasive lobular
PL21	73	highly pos/low pos	2+	30%	n.i.	invasive ductal
PL23	84	moderately pos/ low pos	3+	n.i.	n.i.	invasive ductal
PL24	63	neg/neg	3+	n.i.	bone	invasive ductal
PL25	81	moderately pos/neg	pos	60%	cutis	invasive ductal

ER, estrogen receptor; PR progesterone receptor; Her2, human epidermal growth-factor receptor 2; n.i., no information available; pos, positive; neg, negative

### FACS: Cell sorting for CSC markers CD44^+^/CD24^-/low^ and ALDH1^+^

The isolated cells were sorted within a median time of 8 days after collection of pleural effusion aspirates for cancer stem cell markers CD44^+^/CD24^-/low^ and ALDH1^+^. Cells from subpopulations were seeded for sphere formation assay into ULA flasks with a median cell number of 2*10^4^ cells (range 500–6.5*10^5^ cells). Additionally, cells were collected for DNA extraction with a median cell number of 1.4*10^5^ cells (range 1.7*10^3^–1.2*10^6^ cells).

**Fig 1:**
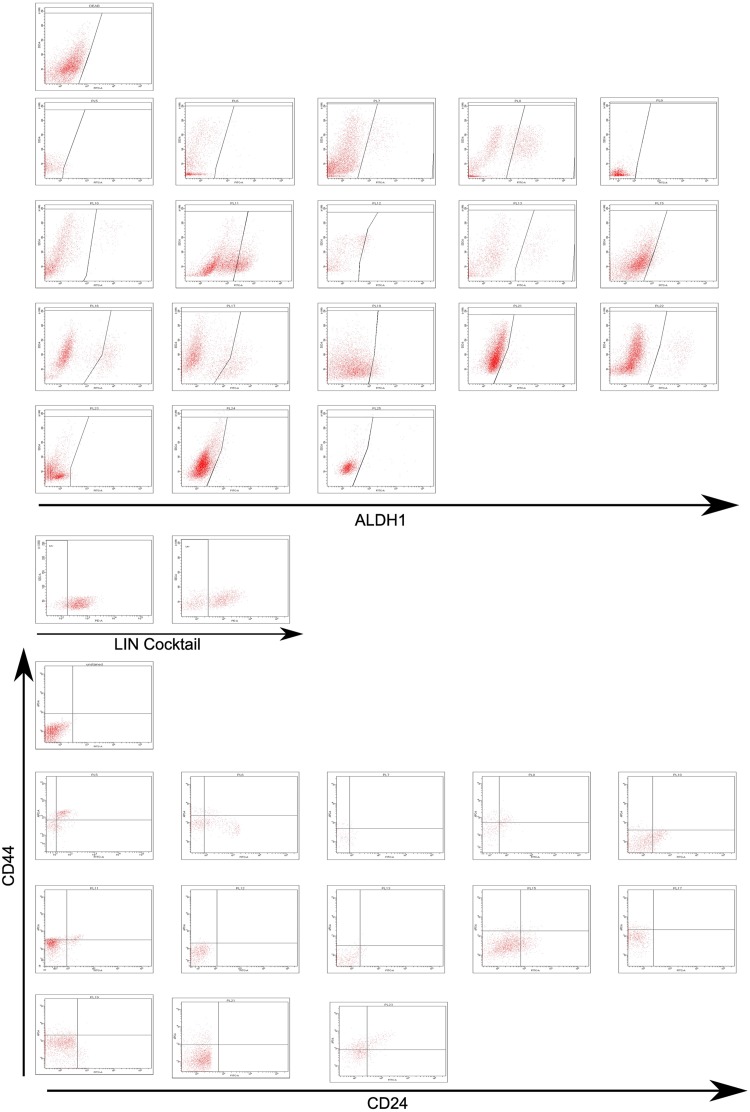
Expression of cancer stem cell markers ALDH1 and CD44^+^/CD24^-/low^ in malignant pleural effusions of metastatic breast cancer patients. (A) Aldefluor assay measuring ALDH1 expression in individual pleura samples. (B) FACS dot plots of CD44^+^/CD24^-/low^ staining. First plots show settings for linage cocktail staining, positive cells were depleted. Both images A and B also include a representative plot of an unstained sample.

The expression of ALDH1 was assessed in 18 pleural effusion samples. As shown in Fig 1 as well as in Table 2 the frequency of ALDH1 positive cells varied in the pleural effusion samples with a median percentage of 2.7 (Range: 0.4–22.5%).

**Table 2:** Summarized results from sphere formation of unsorted cells, CD44^+^/CD24^-^ and ALDH1^+^ subpopulations from 18 analyzed pleural samples.

Sample ID	spheres from unsorted cells	CD44^+^/CD24^-^ (%)	spheres	ALDH1^+^ (%)	spheres
PL5	No	7.7	No	0.6	No
PL6	Yes	11.8	No	0.5	No
PL7	Yes	14.0	No	2.9	No
PL8	Yes	15.1	No	14.7	Yes
PL9	Yes	nc	nc	0.5	No
PL10	Yes	3.2	Yes	2.7	Yes
PL11	Yes	16.4	Yes	22.5	Yes
PL12	No	2.1	No	8.3	No
PL13	Yes	4.1	Yes	9.3	Yes
PL15	No	1.9	No	2.1	No
PL16	Yes	nc	nc	12.6	No
PL17	Yes	10.5	No	14.5	No
PL19	Yes	5.3	Yes	2.2	No
PL21	Yes	3.3	No	1.1	No
PL22	Yes	nc	nc	5.8	Yes
PL23	No	27.7	No	0.4	No
PL24	Yes	nc	nc	0.5	nc
PL25	Yes	nc	nc	0.6	nc

ALDH1, Aldehyde dehydrogenase 1

Yes: spheres appeared in primary sphere culture and could be passaged on;

No: no spheres evolved;

nc: not enough cells for cell sorting; sphere formation assay was not performed

Limited by the low cell numbers of some samples, the expression of CD44^+^CD24^-/low^ was evaluated in 13 out of 18 (72%) pleural effusions. The median percentage of CD44^+^CD24^-/low^ was 6.5% (Range: 1.9%-27.7%) as shown in Fig 1 and Table 2.

### Culturing of mammospheres from unsorted and sorted cells of pleural effusions

**Fig 2:**
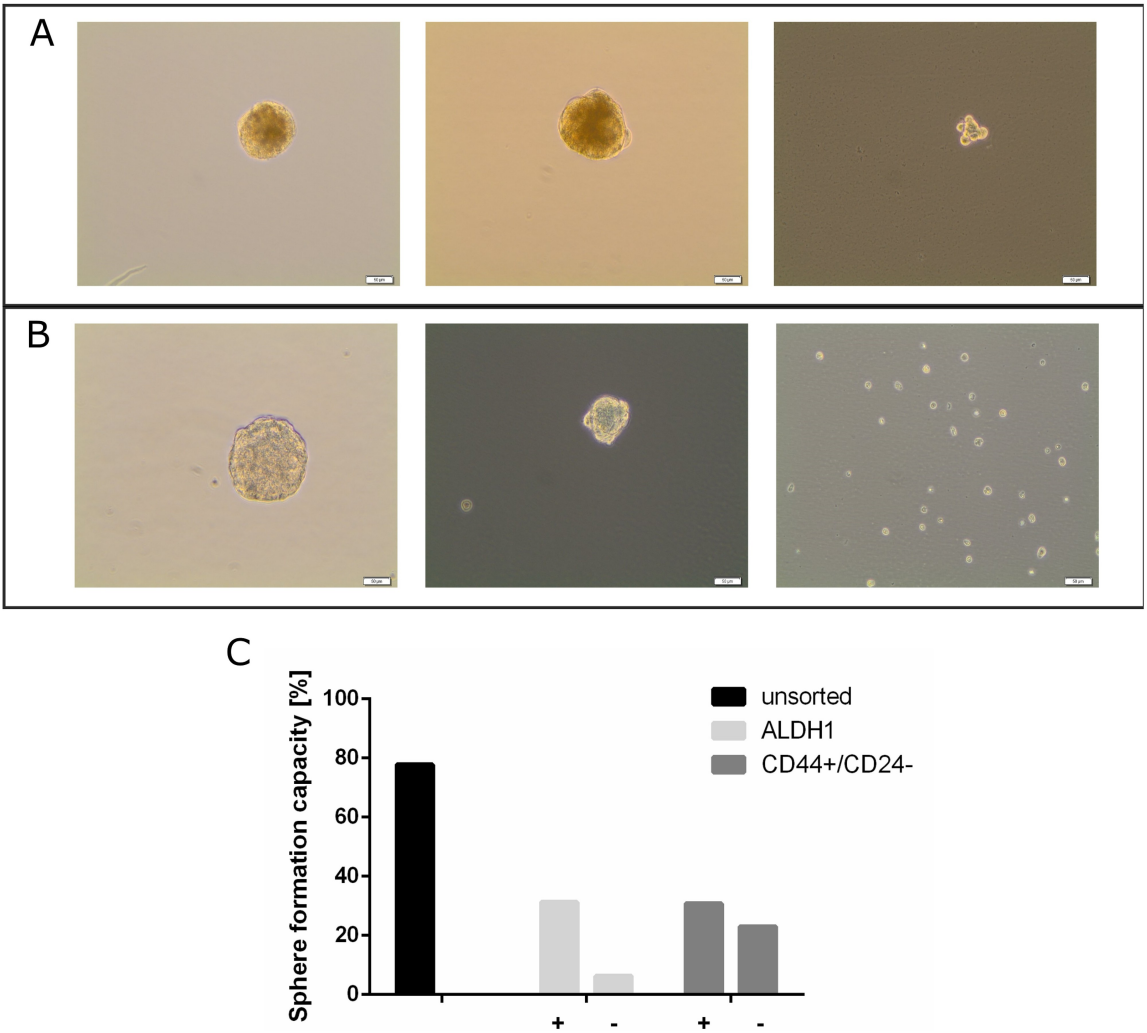
Sphere formation assay. Representative pictures from mammosphere assay. (A) PL11, left sphere from unsorted cells, middle sphere from CD44^+^/CD24^-/low^ subpopulation, right cells from CD44^-^/CD24^-^ subpopulation. (B) PL13, left sphere from unsorted cells, middle sphere from ALDH1^+^ subpopulation, right cells from ALDH1^-^ subpopulation. Scale bar 50 μM. (C) Summary of sphere formation efficiency of all pleura samples with their different subpopulations.

The protocol for cell isolation was improved after sample 13. Optimization steps included filtration of the fresh pleural effusion through a wide mash and another filtration with a 70 μm strainer after the lysis step. The spheroid culture was performed to assess primary sphere forming capacity of unsorted and sorted cells. Overall, a higher success rate of primary spheres was achieved with unsorted cells (14 out of 18; 77.8%) compared to sorted subpopulations. Representing pictures of primary mammospheres generated from PL 11 and 13 are shown in Fig 2A and 2B.

Subpopulations obtained after the cell sorting were placed in non-differentiating media in non-adherent culture flasks or plates to determine the primary sphere formation efficiency. The ability of sphere formation is summarized in Table 2. Both putative breast cancer stem cell subpopulations expressing the ALDH1 or the CD44^+^CD24^-/low^ phenotype, showed similar sphere formation efficiency. In detail, we were able to generate primary spheres from four out of 13 (30.77%) subpopulations with the stem cell phenotype CD44^+^CD24^-/low^ and five out of 16 (31.25%) subpopulations with high expression of ALDH1. Three samples (13.64%) generated spheres under all three conditions including unsorted cells, CD44^+^CD24^-/low^, and ALDH1 positive cells. In some pleura samples the negative subpopulations also formed spheres. The CD44^-^/CD24^-/low^ subpopulations showed higher sphere formation efficiencies (3 out of 13; 23.08%) than the ALDH1 negative subpopulations (1 out of 16; 6.25% Fig 2C).

### Copy number profiling

In order to genetically characterize the different tumor samples and cell fractions, we performed copy number analysis by employing low-coverage whole genome sequencing (SCNA-seq, somatic copy number alterations) of nine pleura samples from eight patients. For all cases, DNA from paraffin-embedded primary tumors or metastases was available for comparisons of the genome wide copy number status inferred from the observed read counts across the genome [27]. A total of five from nine analyzed primary pleura samples (55%) showed aberrant copy number profiles similar to the respective primary tumor. Based on the analytical sensitivity of the SCNA-Seq, at least 10% of cells originated from a tumor in these samples [27]. Due to low DNA yields after cell sorting, only six ALDH1^+^ and three CD44^+^/CD24^-/low^ cell fractions, respectively, could be analyzed. Interestingly, except for one case (PL21) all analyzed ALDH1^+^ cells neither mirrored any of the respective alterations from the primary tumor, nor showed novel changes. In contrast, all samples showed balanced copy number profiles. As for the CD44^+^/CD24^-/low^ cell fractions, one showed tumor-specific CNAs, while the other two were balanced.

**Fig 3:**
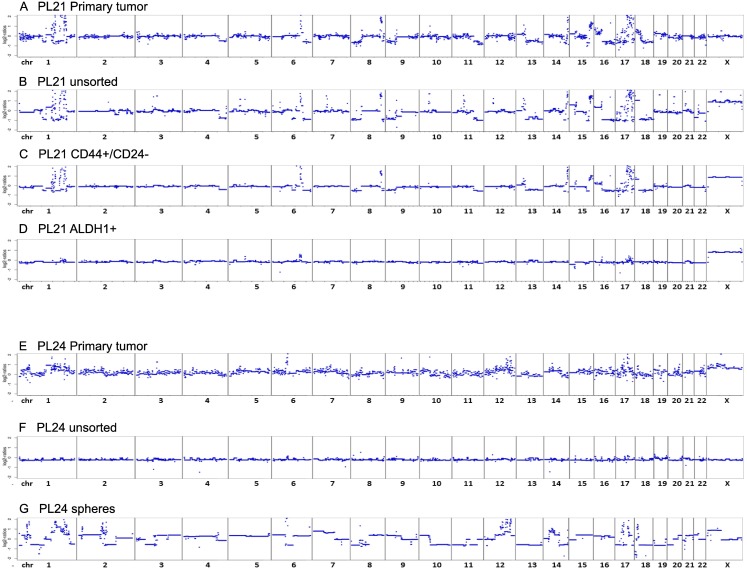
Copy number profiles. Primary tumors of PL21 (A) and PL24 (E), the cultivated unsorted cells before cell sorting (B) (F), CD44-positive cell fraction of PL21 (C) and the ALDH1^+^ cell fraction of PL21 (D), respectively, after FACS cell sorting. PL24 long-term cultivation of unsorted spheroids (G). Depicted are segmented log2-ratio plots of the genome. The X-axis indicates the chromosome and Y-axis indicate the log2-ratios. Regions with log2 ratios > 2 indicate gain of chromosomal material and those regions with log2 ratios < 2 indicate loss of chromosomal material.

Two cases (PL21 and PL24) will be discussed in more detail. The primary tumor of PL21 showed a variety of copy number alterations (CNAs), including losses at chromosomes 4q, 8p, 9p, 11q,or 13q that are frequently observed in breast cancer [28] (Fig 3A, S1 Fig). In addition, many high level gains at chromosomes were present of which many appeared as high-level focal amplifications (size <20Mb, log2-ratio > [29]) located at chromosomes 1q, 6, 7, 8, 10, 13q, 14q, 15q, 17, and 18p. Most of the CNAs from the primary tumor were also observed in the corresponding unsorted pleura sample (Fig 3B, S1 Fig) indicating a common origin. However, the unsorted pleura sample showed additional focal amplification on chromosomes 7, 11, 12, and 20. The CD44^+^ cell fraction showed a highly consistent copy number profile with both, the primary tumor and the unsorted pleura sample (Fig 3C). It is of note that the CD44^+^ cell fraction resembled the primary tumor to a higher degree, since both samples lack the focal amplifications seen in the unsorted of pleura sample. The ALDH1^+^ cell fraction showed only a few focal amplifications on chromosomes 6, 16 and 17 with lower amplitudes (Fig 3D, S1 Fig) indicating a low tumor fraction. Interestingly, some of the high level gains (on chromosome 6, 16 and 17) were found in all samples while other high level amplifications with similar log2-ratios (on chromosome 1, 8, 14, 21) were only present in some samples. For example, prominent focal amplifications of chromosome 1 could be detected in the primary tumor, the unsorted pleural sample and in the CD44^+^ cell fraction. In contrast, no focal amplifications of chromosome 1 were found in the ALDH1^+^ fraction. These results indicate that the ALDH1^+^ fraction contains a subpopulation of putative breast cancer cells with a different genetic profile.

In contrast, the unsorted pleura sample PL24 presented an almost balanced copy number profile (Fig 3E). This can most likely be attributed to a high background of normal cells. However, after cultivation of unsorted cells in sphere forming media, tumor cells were highly enriched and the copy number profile reflected aberrations of the primary tumor. Despite a strong background noise in the primary tumor sample due to low DNA quality after paraffin embedding, we were able to reconstruct the major CNAs of the tumor sample in the sphere culture (Figs 3E+3G and 4A). Again we observed a number of high level focal amplifications on chromosomes 1, 12, 14, 17 (including *ERBB2*), and 18. Due to limited material we were unable to establish copy number profiles for CD44^+^ and ALDH1^+^ cells.

**Fig 4:**
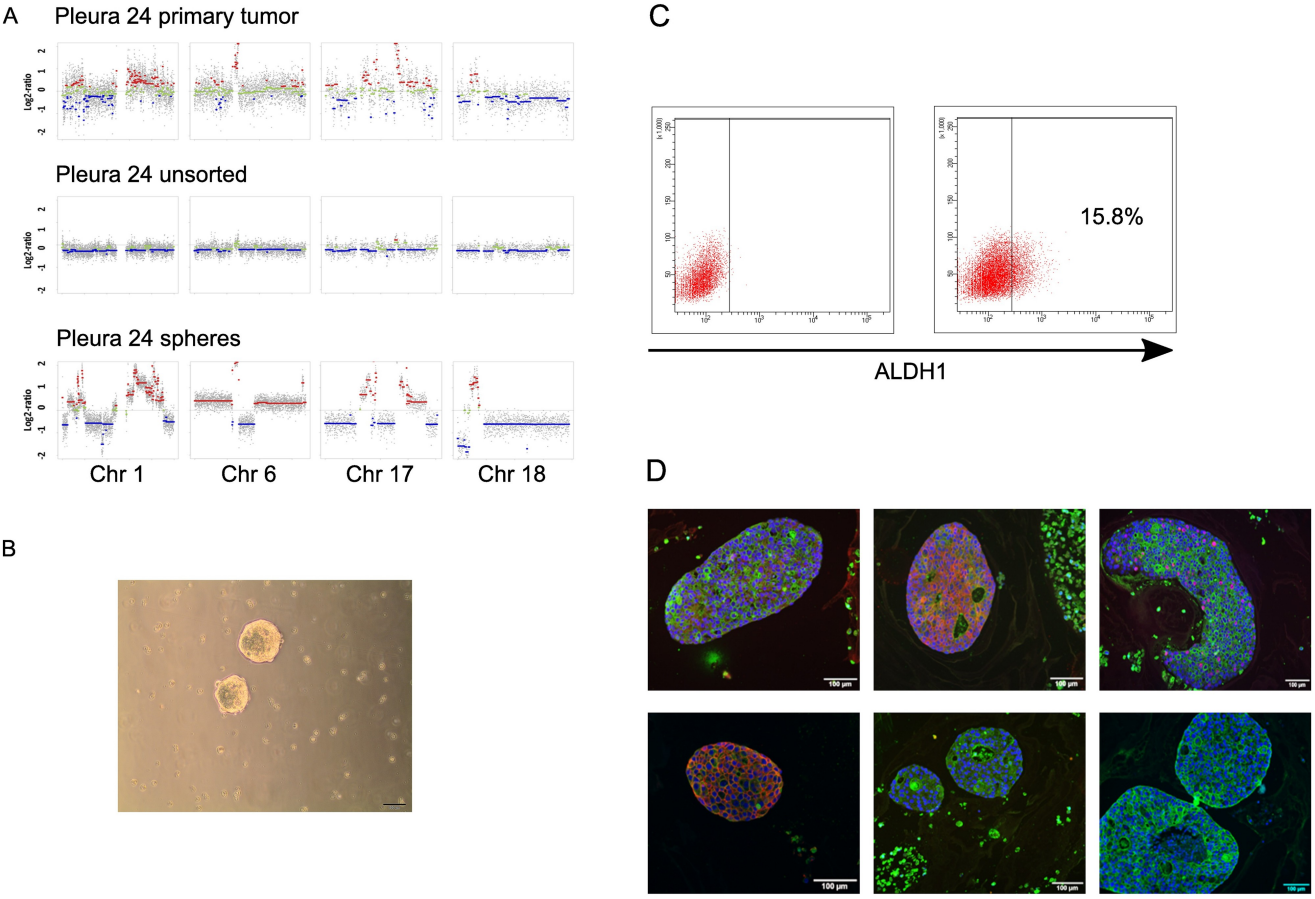
Characterization of PL24. (A) Selected copy number plots of PL24. Depicted are segmented log2-ratio plots of chromosomes 1, 6, 17 and 18. (B) Representative picture of spheres in culture. (C) FACS dot plots of PL24 passage 20 left control with DEAB and right Aldefluor assay staining. (D) Immunofluorescent staining of FFPE PL24 spheres I ALDH1 (red) CK (green), II CD44 (red) CK (green), III Ki67 (red) CK (green), IV HEA (red) CK (green), V Vim (red) CK (green), VI Her2neu (green). All slides were counterstained with Dapi (blue). Measuring bar: 100 μm.

### Established cell line from pleura effusion aspirate

Only in one case, the unsorted pleura samples PL24, we were able to establish a cell line, and cells could be passaged continuously. This cell line can still be cultured (Fig 4). A contamination with other cell lines in culture was excluded with an STR analysis with DNA from the primary tumor serving as a control (data not shown). In this particular case, the spheroid formation started by forming tubular structures in the first 3–5 days and continued by formation of round shaped spheroids. The spheroids became progressively larger. Interestingly, cells were not able to attach under adherent culture conditions using FBS.

The spheroid cell line was further characterized with flow cytometry and immunofluorescence for CSC and EMT marker expression. FACS analysis of LT24 spheres passage showed a significant increase of the CSC marker ALDH1 from an initial 0.5% to 15.2% at passage 20. The ALDH1 expression was also confirmed with immunofluorescent staining of FFPE spheroids. Initially, flow cytometry analysis of CD44^+^/CD24^-/low^ was not done due to low cell amount, but spheroids were highly positive of CSC marker CD44 with immunofluorescent staining. Ki67 staining of 25% suggested an aggressive behavior due to high proliferation rate. A positive expression of human epithelial antigen (HEA) and pan-cytokeratin (CK) and absence of Vimentin staining indicated an epithelial phenotype. The Her2neu overexpression of the primary tumor could be confirmed with an intensive Her2neu positive staining of the spheres. The underlying gene amplification of *ERBB2* leading to over expression of Her2neu was also evident in the copy number profiles pictured in Figs 3E–3G and 4A.

## Discussion

Our study represents to our knowledge the first study to prospectively evaluate pleural effusions from metastatic breast cancer patients as a source for enrichment of cancer stem cells and further molecular characterization by low coverage sequencing.

Pleural effusion aspirates provide a unique biological tool to study the formation and biology of metastasis. The occurrence of malignant pleural effusions is correlated with poor prognosis of tumor disease [30]. This biological source is a suitable model to study CSC in circulation and it has been already used for various applications [31]. In our study, culturing tumor cells from unsorted pleural effusions under non-adherent culture conditions was successful in 77.8% of the patient samples. This success rate is similar to other studies, where 42–73% of spheroid cultures from pleural effusion aspirates could be initiated [32–34]. However, after sorting for putative breast cancer stem cell markers, the spheroid formation efficiency in these subpopulations was diminished. Besides an additional stressor on sensitive primary cells during the cell sorting process, paracrine or autocrine signal molecules from other cells contained in pleural effusion are lost after sorting [35]. Recent published literature also emphasizes the significance of the pleural fluid itself where cytokines and chemokines enhance proliferation and migration [36].

With the focus of sequencing putative breast cancer stem cells in order to find new biomarkers, we investigated differences in copy number profiles between the primary tumors compared to metastatic cells isolated from the pleural effusion. While we were able to analyze all unsorted pleura samples and to recover tumor-specific alterations in 55% of all cases, cell sorting of subpopulations did not yield sufficient cells for copy number analysis for the majority of samples. This limitation was still present, although the entire effusion sample with a maximum of 1500 mL was processed for sorting and all sorted cells were used for sequencing.

Moreover, most subpopulations showed a balanced profile. Depletion with lineage cocktail and stringent gate setting strategy could not completely exclude all normal cells. These results may indicate insufficient CSC marker relevance or a minimal concentration of CSC after isolation along with an insufficient sensitivity of the sequencing method. The lack of effectiveness of common CSC marker expression has been discussed in literature [37–39] and particularly with regard to tumor heterogeneity the demand for new biomarkers is still prominent.

The relevance for the need of a sufficient amount of tumor cells was reflected in PL24. The unsorted primary cells did not show any aberration, but after *in vitro* cultivation, a CSC population could be enriched and genomic aberrations of these cells could be detected. These results also implicated the importance of appropriate CSC marker expression. PL21 showed a focal amplification in chromosome 6 and 17 of the primary tumor, which was also seen in unsorted cells and in the CD44^+^/CD24^-/low^ subpopulation. This aberration was also observed in the ALDH1^+^ cell fraction, although with a much lower amplitude indicating a lower amount of tumor cells in this subpopulation. Low-coverage sequencing requires 5–10 malignant cells within 100 pleural effusion cells in order to detect possible aberrations [27]. It is also important to mention that cells with a balanced genomic profile could also be tumor associated cells such as macrophages, which support disseminated tumor cells [40]. Taken together our results indicate the need of more effective enrichment methods. A higher specificity could also be achieved by sequencing a specific gene panel. For example, Chen et al. analyzed cells from pleural effusions by sequencing frequent mutations of lung cancer disease [41]. Taken together, these results emphasize the need of either a long term CSC enriched cultures or high number of enriched cancer stem cells per patient in order to generate sufficient material, which can be used to comprehensively analyze the biology of CSC at the molecular and functional level. We show here that this is rarely a case in an unselected population of patients with pleural effusions. Our results suggest therefore that conclusions drawn so far were based on a highly selected population of breast cancer patients.

In conclusion, we were able to enrich and analyze putative cancer stem cells from pleural effusions from an unselected population of patients. However, whole genome sequencing analysis of CSC sorted cells is substantially limited by the amount of tumor cells and insufficient specificity of CSC markers. Therefore, in most cases the use of a more sensitive high-resolution method and additional CSC markers are necessary in order to detect relevant genomic changes. Nevertheless, conclusions from our study are an important basis for future validation studies.

## Supporting information

S1 Fig Selected copy number plots for chromosomes 6 and 17. Depicted are segmented log2-ratio plots chromosome 6 and 17. The X- and Y-axes indicate the chromosome and the log2-ratios, respectively. Primary tumor, the unsorted primary cells, and the CD44-positive cells show very consistent copy number profiles. The high level gains on the long arm of chromosomes 6 and 17, respectively can also be observed in the ALDH-positive cell fraction, although with a much lower amplitude indicating a lower amount of tumor cells.
(PDF)
