# Supplementary material for: Genetic profiling of putative breast cancer stem cells from malignant pleural effusions
Source: PLoS One. 2017 Apr 19;12(4):e0175223. doi: 10.1371/journal.pone.0175223 (PMC5396869; doi:10.1371/journal.pone.0175223)
Supplement: S1 Fig — Depicted are segmented log2-ratio plots chromosome 6 and 17. The X- and Y-axes indicate the chromosome and the log2-ratios, respectively. Primary tumor, the unsorted primary cells, and the CD44-positive cells show very consistent copy number profiles. The high level gains on the long arm of chromosomes 6 and 17, respectively can also be observed in the ALDH-positive cell fraction, although with a much lower amplitude indicating a lower amount of tumor cells. (PDF) [file pone.0175223.s001.pdf]

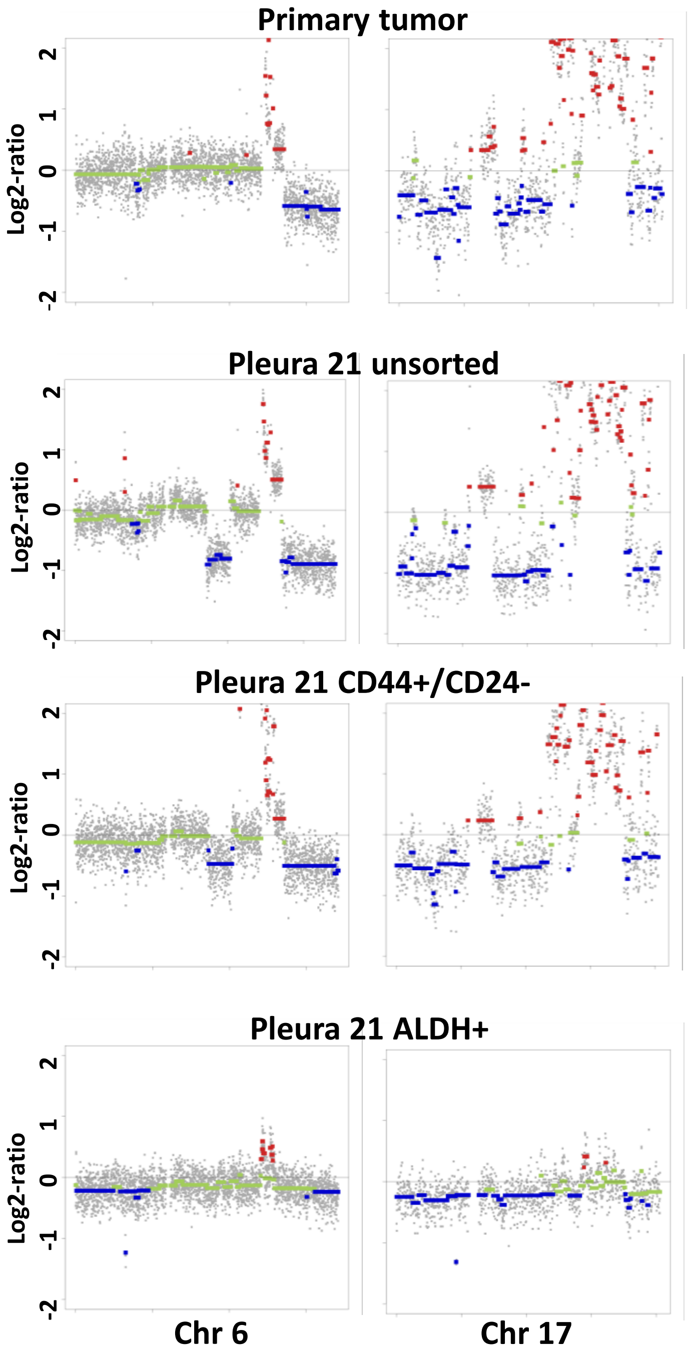

Supp Figure 1. Selected copy number plots for chromosomes 6 and 17.

Depicted are segmented log2-ratio plots chromosome 6 and 17. The X- and Y-axes indicate the chromosome and the log2-ratios, respectively. Primary tumor, the unsorted primary cells, and the CD44-positive cells show very consistent copy number profiles. The high level gains on the long arm of chromosomes 6 and 17, respectively can also be observed in the ALDH-positive cell fraction, although with a much lower amplitude indicating a lower amount of tumor cells.
